# Inducible Displacement of Cementless Femoral and Tibial Components Throughout Weight-bearing Flexion

**DOI:** 10.1016/j.artd.2025.101723

**Published:** 2025-06-04

**Authors:** Rebecca A. Hext, Jordan S. Broberg, James L. Howard, Brent A. Lanting, Matthew G. Teeter

**Affiliations:** a Department of Medical Biophysics, Schulich School of Medicine & Dentistry, Western University, London, ON, Canada; b Division of Orthopaedic Surgery, Schulich School of Medicine & Dentistry, Western University, London, ON, Canada

**Keywords:** Cementless total knee arthroplasty, Inducible displacement, Radiostereometric analysis, Knee flexion

## Abstract

**Background:**

Cementless fixation for total knee arthroplasty has been increasing following advancements in implant designs. The use of cementless designs has been supported through longitudinal radiostereometric analysis (RSA) studies; however, few studies have used inducible displacement exams to assess fixation throughout flexion. Our primary aim was to assess patterns and locations of inducible displacement throughout flexion.

**Methods:**

Participants (n = 24) received a fixed-bearing, cruciate-retaining cementless implant. At 1-year postoperation, participants underwent a supine RSA exam and standing RSA exams at 0°, 20°, 40°, and 60° of knee flexion. Inducible displacements were reported as maximum total point motion and as 3-dimensional translations at points of interest.

**Results:**

Inducible displacement of the tibial component increased with knee flexion angle and was 1.129 ± 0.644 mm at 0°, 1.181 ± 0.462 mm at 20°, 1.526 ± 0.386 mm at 40°, and 1.648 ± 0.461 mm at 60°. Inducible displacement of the femoral component increased with knee flexion angle and was 0.704 ± 0.364 mm at 0°, 0.839 ± 0.458 mm at 20°, 1.011 ± 0.451 mm at 40°, and 1.203 ± 0.708 mm at 60°. The strongest correlations between 3-dimensional translation and knee flexion angle were at the stem tip for the tibial component, and anterior flange tip for the femoral component.

**Conclusions:**

Inducible displacement measurements increased with knee flexion angle. At 0°, both components had values consistent with well-fixed components. The locations of maximal displacements support these components are well-fixed and demonstrate that inducible displacement throughout flexion is due to mechanical loading.

## Introduction

The use of cementless fixation for primary total knee arthroplasty (TKA) has been increasing following advances in implant design [1,2]. Additive manufacturing has enabled the creation of complex porous metal surfaces that may provide an advantage for long-term biological fixation [[3], [4], [5]]. Multiple clinical and radiostereometric analysis (RSA) studies have supported their use by showing sufficient fixation of these components [[5], [6], [7], [8], [9]]. However, further investigation of cementless implants during activities of daily living is required, especially due to an increase in younger, more active patients getting the procedure [10,11].

Experimental and computational methods have previously been used to assess modern cementless TKA designs during activities of daily living [[12], [13], [14], [15]]. RSA is a well-established tool for measuring implant migration, usually over a series of multiple visits spanning 2 years, which can be used to predict long-term component loosening [16,17]. RSA can also be used to measure inducible displacement, defined as the magnitude of displacement between loaded and unloaded exams at a single timepoint [18,19]. Measures of inducible displacement can provide instantaneous insight into how well fixed the component is and can be used as a tool to examine knee loading [20]. While the implant migration of modern cementless TKA designs has been well studied, there is a limited number of studies examining the inducible displacement of these devices [20,21]. Fewer still investigate inducible displacement over a range of flexion angles [22].

Appropriate component size relative to the underlying bone is important for TKA longevity [23,24], especially in cementless TKA where loads are transferred directly from the implant to the bone [15]. The tibial baseplate may be undersized to allow greater rotational freedom, or if a perfect fit for the patient is not possible [15,25]. The femoral component may be undersized if the bone size is between implant sizes and the smaller size is used. It may also be undersized if the femoral component is either anteriorized or posteriorized based on the reference technique used. With an undersized femoral component, notching of the anterior femoral cortex may occur. Notching may compromise the cortical bone and concentrate stress at the proximal end of the femoral component and has been investigated due to the risk of periprosthetic fractures [24,26,27]. Undersizing of the tibial baseplate has been investigated in finite element studies of the bone–implant interaction, or in clinical RSA migration studies [15,25]. However, the impact of both tibial and femoral component sizing on fixation throughout flexion has not been investigated.

Cementless TKA has been increasing and due to its reliance on bone ingrowth, further investigation is required into the impact that weight-bearing flexion and component size have on implant fixation. Therefore, the goal of this study was to assess inducible displacement of cementless femoral and tibial TKA components at multiple knee flexion angles at 1-year postoperation using RSA. Our specific objectives were to (1) assess if the pattern of inducible displacement changes depending on the knee flexion angle, (2) assess the maximal location of inducible displacement using specific points of interest on the implant component (fictive points), and (3) determine if the magnitude or pattern of inducible displacement changes depending on knee flexion angle between patients with or without femoral notching and with or without tibial baseplate underhang.

## Material and methods

A prospective RSA cohort study investigated the effect of gap balancing vs measured resection surgical technique on cementless tibial baseplate migration [13]. The surgical techniques showed no differences in maximum total point motion (MTPM), translations, and rotations with respect to tibial or femoral component migration from 2 weeks to 1 year postoperation. This is an analysis of data acquired as part of that study that has not previously been analyzed. All participants scheduled for a TKA between September 2017 and May 2018 were screened and only eligible participants were approached for recruitment [13]. A total of 48 participants were recruited to the study, and 9 were excluded due to requiring cement fixation (n = 6), surgery cancellation (n = 2), or RSA beads not being inserted (n = 1) [13]. Therefore, there were 39 participants available for inclusion in the study; however, due to occluded markers in the RSA flexion exams, 24 participants were included in the current analysis. There were 9 men and 15 women with a mean age at surgery of 63 years (range, 48-73 years) and a mean body mass index of 34.7 kg/m^2^ (range, 25.3-49.1 kg/m^2^).

In the current analysis, the data from both groups were pooled. Ethics approval was obtained from our institutional ethics review board (REB #109486) and all subjects provided written informed consent before participation. Subjects who were a minimum age of 18 years and had a primary diagnosis of osteoarthritis were included. Subjects were excluded based on a diagnosis of inflammatory arthritis, had prior knee surgery, were or planned on becoming pregnant, had a cognitive impairment preventing completion of questionnaires, a neuromuscular disorder preventing completion of a functional walking test, an inability to understand English, a history of alcoholism, or were greater than 75 years of age.

Two high-volume arthroplasty surgeons performed operations between October 2017 and June 2018. All subjects received a fixed-bearing, cruciate-retaining beaded periapatite-coated cementless femoral component and a pegged highly porous cementless tibial baseplate with a condylar-stabilizing tibial insert (Triathlon Tritanium, Stryker, Mahwah, NJ). With this implant design, the surgeons retained the posterior cruciate ligament. Up to 8 tantalum beads were placed in both the proximal tibia and distal femur intraoperatively to enable RSA evaluation. Postoperative protocols were identical for all subjects.

A conventional supine RSA exam was acquired at 2 weeks, 6 weeks, 3 months, 6 months, and 1 year postoperation using a biplanar calibration cage (RSA Biomedical, Umea, Sweden). A standing exam was also acquired at 1 year postoperation using a uniplanar calibration cage (RSA Biomedical) with the subject’s body weight equally distributed between limbs and hand supports for balance. The subjects then performed stationary weight-bearing exams at multiple knee flexion angles measured with a goniometer (20°, 40°, and 60°) with the weight equally distributed between limbs.

**Figure 1:**
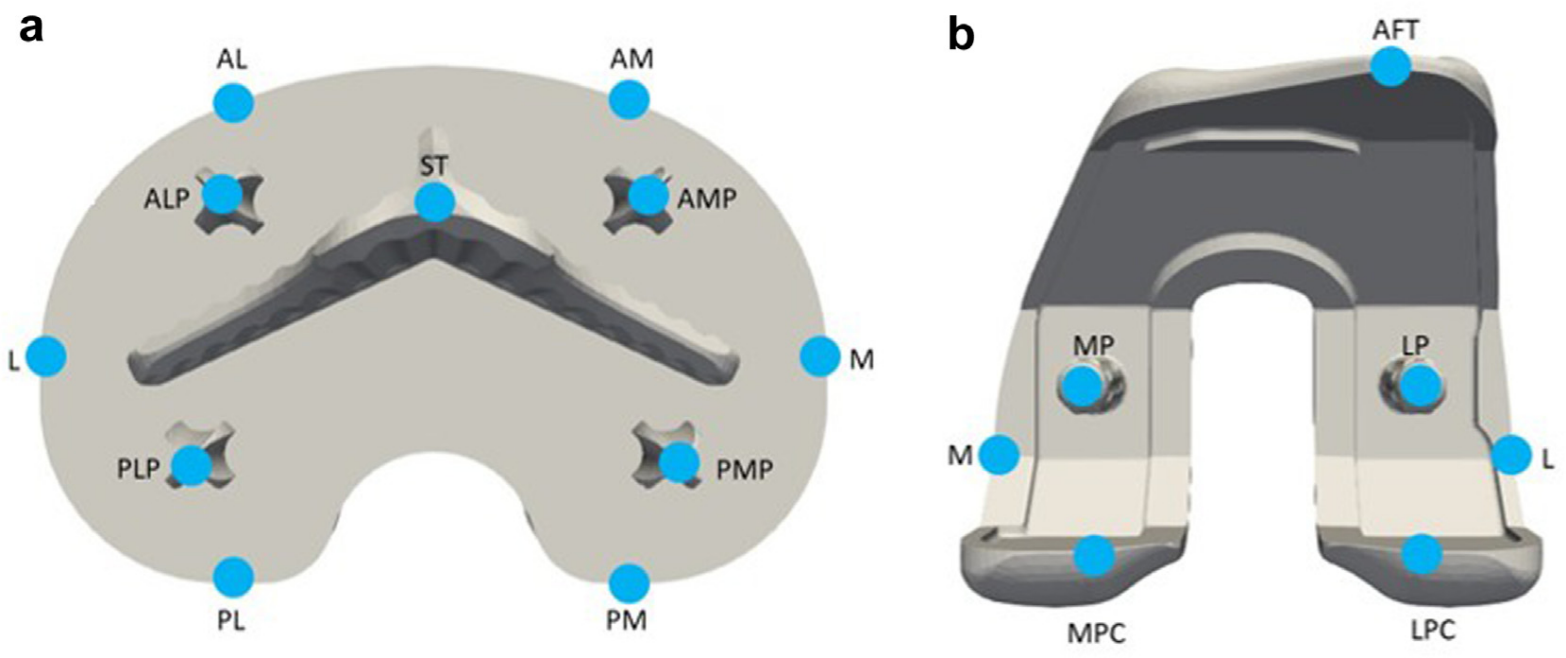
Fictive points placed around the tibial (a) and femoral (b) component. (a) Inferior view of a 3D model of the right tibial component showing the location of the fictive points (blue spheres). (b) Superior view of a 3D model of a right femoral component showing the location of the fictive points (blue spheres). M, medial; PMP, posteromedial peg; PM, posteromedial; PL, posterolateral; PLP, posterolateral peg; L, lateral; ALP, anterolateral peg; AL, anterolateral; ST, stem tip; AM, anteromedial; AMP, anteromedial peg; AFT, anterior flange tip; LP, lateral peg; L, lateral; LPC, lateral posterior condyle; MPC, medial posterior condyle; M, medial; MP, medial peg.

Model-based RSA software (RSACore, Leiden, Netherlands) was used to find the 3-dimensional (3D) poses of the implant models (Stryker) and to determine the location of the tantalum markers representing the pose of the femur and tibia bones. The actual flexion angle of each RSA examination was determined from the pose of the femoral component relative to the tibial component. Model-based RSA was then used to measure inducible displacement, defined as implant motion between the supine exam and each loaded exam at flexion. Inducible displacement measurements were reported as MTPM and as 3D translations at different points of interest, called fictive points, placed around both the femoral and tibial component (Fig. 1). Double examinations were not performed for this cohort due to ethics radiation requirements in 2018 when this study was performed. A previous study at our facility used RSA for the same implant design and found the repeatability for artifactual migration was 0.195 mm for MTPM, and for apparent MTPM, repeatability was 0.198 mm [4]. The RSA coordinate system is based on migration of the implant in the right-hand side of the patient and is defined with positive translation as proximal translation in the y-axis, medial translation in the x-axis, and anterior translation in the z-axis [17]. Positive rotations were defined as anterior tilt about the x-axis, internal rotation about the y-axis, and valgus rotation about the z-axis [28]. Measurements were adjusted for a right-sided implant.

A subgroup analysis of the femoral components was performed to determine if femoral notching or overhang influenced the inducible displacement. Femoral notching was determined by the presence of an anterior notch in the femoral cortex. Overhang was determined by a gap between the surface of the femur and the implant at the anterior flange tip. Lateral radiographs were used to divide patients into 3 subgroups: notched if there was femoral notching present (n = 5), overhang if there was femoral overhang present (n = 8), or normal if neither notching nor overhang was identified in the lateral radiograph (n = 11).

A subgroup analysis of the tibial components was also performed to determine if underhang of the tibial component influenced the inducible displacement. Anterior–posterior radiographs were used to split patients into 2 subgroups: 0/1 sides with underhang if there was no underhang present or if only 1 side had underhang (n = 7), or 2 sides with underhang if there was underhang medially and laterally (n = 17) [29].

For the first objective, a linear mixed-effects model that accounts for repeated measures was used to determine the relationship between inducible displacement and knee flexion angle. Analyses were done for MTPM, as well as 3D translation at each fictive point for both the tibial and femoral components. A linear mixed effects model was used to determine the relationship between inducible displacement MTPM and knee flexion angle for the notched, normal, and overhang groups separately. A linear mixed effects model was used to determine the relationship between inducible displacement MTPM and knee flexion angle for the group with 2 sides of underhang or the group with zero or 1 side with underhang. The level of significance was set at *P* < .05. Statistical tests were conducted using Prism v9.5.1 (GraphPad Software, La Jolla, CA).

This study was conducted in accordance with the ethical and clinical standard of the Institutional Review Board.

## Results

**Figure 2:**
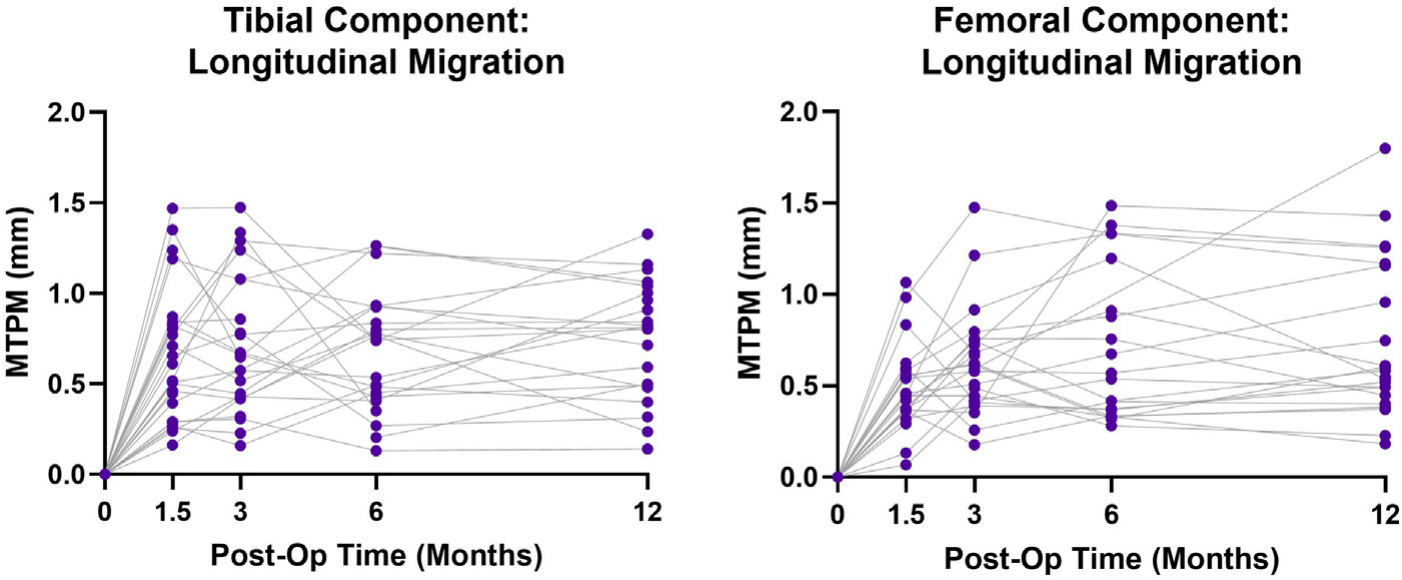
Longitudinal migration of both components from the baseline (2 week) examination to the 1-year examination. Each line represents 1 participant.

To assess implant stability, longitudinal migration was measured with supine examinations (Fig. 2). Mean tibial component MTPM from 6 to 12 months postoperation increased slightly from 0.679 ± 0.332 mm to 0.734 ± 0.327 mm. Mean femoral component MTPM stabilized by 3 months postoperation and decreased from 6 to 12 months postoperation from 0.763 ± 0.474 mm to 0.685 ± 0.368 mm.

Inducible displacement of the tibial component increased with knee flexion angle and MTPM was 1.129 ± 0.644 mm at the 0° exam, 1.181 ± 0.462 mm at the 20° exam, 1.526 ± 0.386 mm at the 40° exam, and 1.648 ± 0.461 mm at the 60° exam (Table 1). The tibial components demonstrated a predominately posterior tilt (negative X axis rotation) throughout flexion.

**Table 1:** Tibial component inducible displacement throughout flexion.

Knee flexion angle	Translations (mm)			Rotations (°)			MTPM (mm)
	X	Y	Z	X	Y	Z	
0°	−0.033 ± 0.114	0.106 ± 0.197	0.165 ± 0.248	−0.063 ± 0.927	−0.040 ± 1.436	−0.093 ± 0.315	1.129 ± 0.644
20°	−0.021 ± 0.115	0.036 ± 0.200	0.050 ± 0.234	−0.536 ± 1.471	−0.160 ± 1.175	−0.035 ± 0.361	1.181 ± 0.462
40°	0.014 ± 0.113	0.076 ± 0.217	0.030 ± 0.332	−0.976 ± 1.973	0.163 ± 1.309	−0.076 ± 0.437	1.526 ± 0.386
60°	−0.017 ± 0.143	0.088 ± 0.280	0.050 ± 0.265	−0.807 ± 2.009	0.047 ± 1.639	−0.015 ± 0.475	1.648 ± 0.461

SD, standard deviation.

Results are reported as mean ± SD for translations in each axis, rotations in each axis, and for MTPM. Positive translations were defined as proximal translation in the y-axis, medial translation in the x-axis, and anterior translation in the z-axis. Positive rotations were defined as anterior tilt about the x-axis, internal rotation about the y-axis, and valgus rotation about the z-axis. Measurements were adjusted for a right-sided implant.

**Figure 3:**
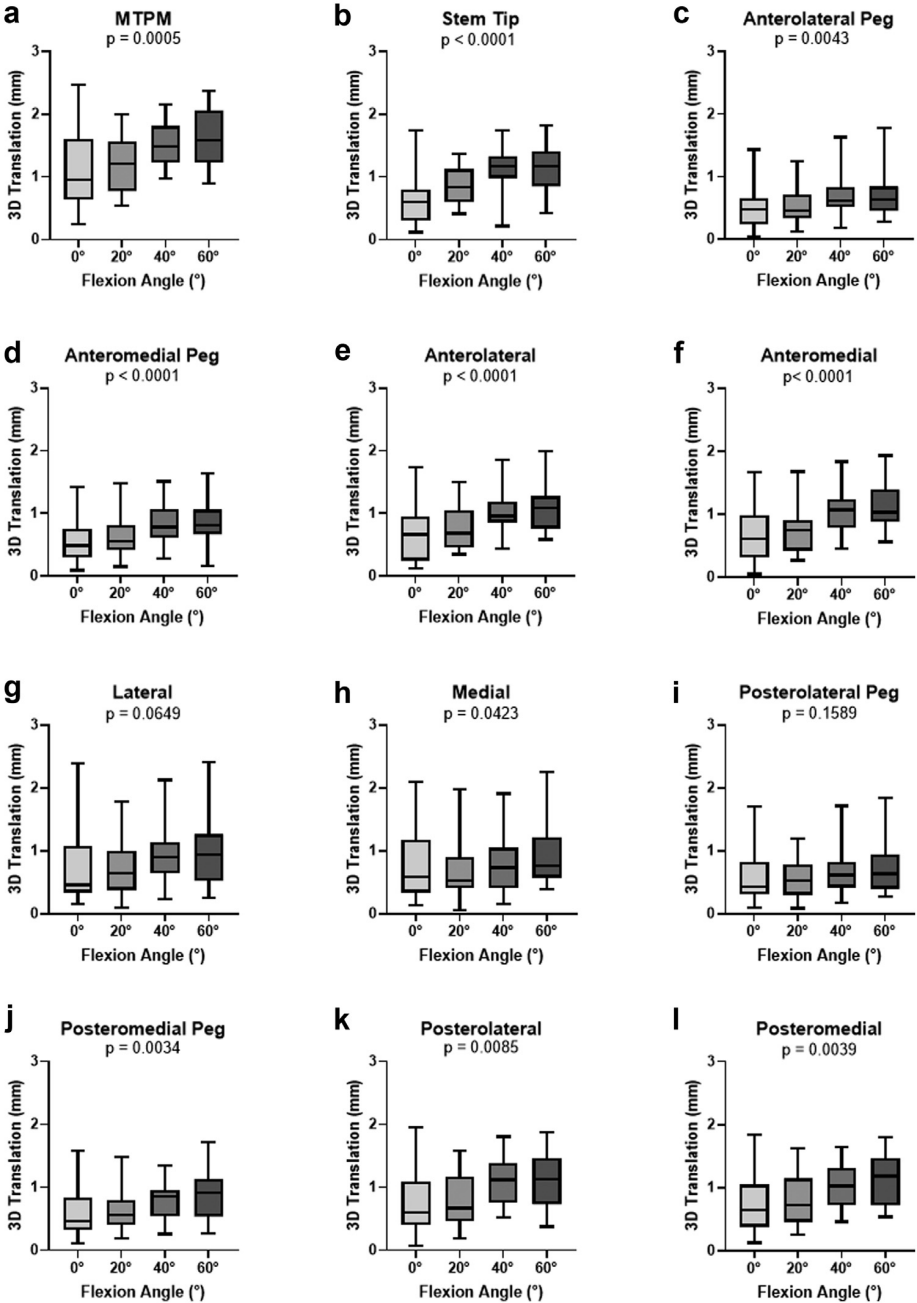
Inducible displacement of the tibial component throughout flexion as MTPM or 3D translations at fictive points. (a) MTPM of the tibial component. (b) 3D translation at the stem tip. (c) 3D translation at the anterolateral peg. (d) 3D translation at the anteromedial peg. (e) 3D translation at the anterolateral position. (f) 3D translation at the anteromedial position. (g) 3D translation at the lateral position. (h) 3D translation at the medial position. (i) 3D translation at the posterolateral peg. (j) 3D translation at the posteromedial peg. (k) 3D translation at the posterolateral position. (l) 3D translation at the posteromedial position.

For the tibial component, the strongest correlations between 3D translation and flexion angle occurred at the stem tip (Fig. 3b, *P* < .0001), anteromedial peg (Fig. 3d, *P* < .0001), anterolateral (Fig. 3e, *P* < .0001), and anteromedial (Fig. 3f, *P* < .0001) fictive points. At 0°, the greatest magnitudes of displacement occurred at the medial (0.811 ± 0.560 mm), lateral (0.747 ± 0.632 mm), posteromedial (0.776 ± 0.485 mm), and posterolateral (0.760 ± 0.499 mm) fictive points. At 60°, the greatest magnitudes of displacement occurred at the stem tip (1.141 ± 0.333 mm), posteromedial (1.132 ± 0.403 mm), and anteromedial (1.127 ± 0.367 mm) fictive points.

**Figure 4:**
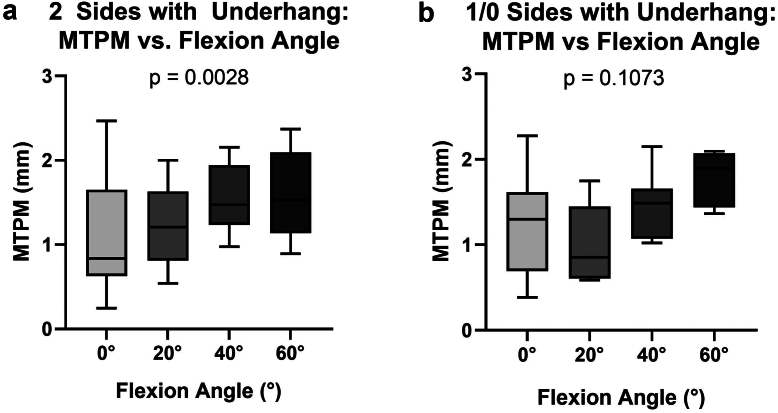
MTPM (in mm) between participants with 2 sides of underhang (a) or only 1 or no sides of underhang (b). There was a significant correlation between MTPM and flexion angle for those with 2 sides of underhang (*P* = .0028).

Components with both medial and lateral underhang had a significant correlation between MTPM and knee flexion angle (Fig. 4a, *P* = .0028). Tibial component MTPM for components with both medial and lateral underhang was 1.076 ± 0.667 mm at 0° and 1.608 ± 0.495 mm at 60°. This is lower than those with 1 or no sides of underhang, which have MTPM of 1.257 ± 0.615 mm at 0° and 1.783 ± 0.328 mm at 60°. Women with 2 sides of underhang had inducible displacement of 1.049 ± 0.677 mm at 0° and 1.681 ± 0.530 mm at 60°, and men with 2 sides of underhang had inducible displacement of 1.115 ± 0.703 mm at 0° and 1.504 ± 0.457 mm at 60°.

**Figure 5:**
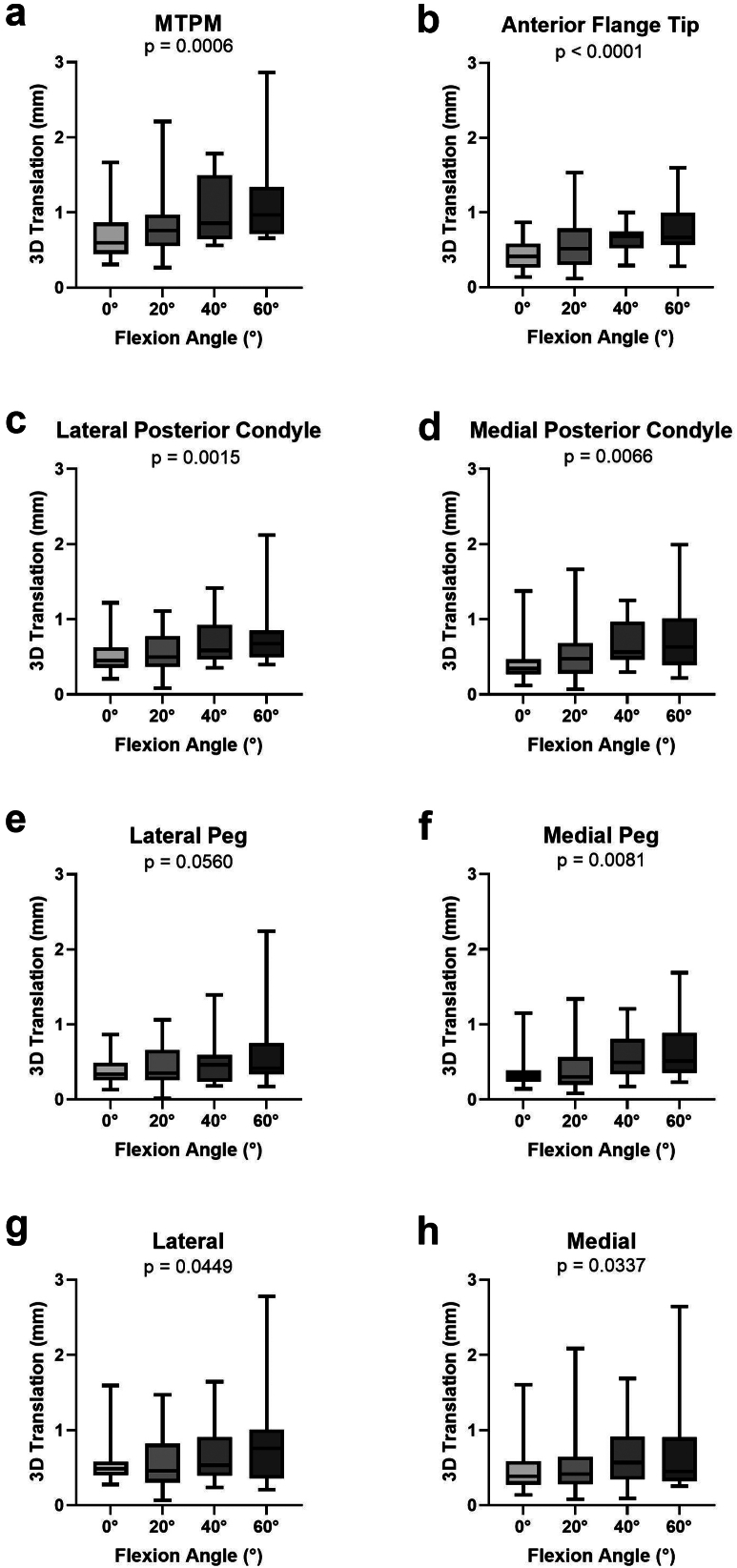
Inducible displacement of the femoral component throughout flexion as MTPM or 3D translations at fictive points. (a) MTPM of the femoral component. (b) 3D translation at the anterior flange tip. (c) 3D translation at the lateral posterior condyle. (d) 3D translation at the medial posterior condyle. (e) 3D translation at the lateral peg. (f) 3D translation at the medial peg. (g) 3D translation at the lateral position. (h) 3D translation at the medial position.

Inducible displacement of the femoral component increased with knee flexion angle and MTPM was 0.704 ± 0.364 mm at the 0° exam, 0.839 ± 0.458 mm at the 20° exam, 1.011 ± 0.451 mm at the 40° exam, and 1.203 ± 0.708 mm at the 60° exam (Table 2). The femoral components had a lateral shift, a small posterior tilt, and external rotation throughout flexion.

**Table 2:** Femoral component inducible displacement throughout flexion.

Knee flexion angle	Translations (mm)			Rotations (°)			MTPM (mm)
	X	Y	Z	X	Y	Z	
0°	−0.039 ± 0.160	−0.010 ± 0.074	0.080 ± 0.314	−0.037 ± 0.366	−0.134 ± 0.565	0.188 ± 0.242	0.704 ± 0.364
20°	−0.117 ± 0.228	−0.001 ± 0.077	0.138 ± 0.359	−0.026 ± 0.517	−0.096 ± 0.622	0.216 ± 0.240	0.839 ± 0.458
40°	−0.085 ± 0.191	0.038 ± 0.107	0.176 ± 0.544	−0.090 ± 0.654	−0.029 ± 0.590	0.334 ± 0.444	1.011 ± 0.451
60°	−0.166 ± 0.295	−0.030 ± 0.142	0.350 ± 0.606	−0.186 ± 0.658	−0.290 ± 0.819	0.090 ± 0.367	1.203 ± 0.708

SD, standard deviation.

Results are reported as mean ± SD for translations in each axis, rotations in each axis, and for MTPM. Positive translations were defined as proximal translation in the y-axis, medial translation in the x-axis, and anterior translation in the z-axis. Positive rotations were defined as anterior tilt about the x-axis, internal rotation about the y-axis, and valgus rotation about the z-axis. Measurements were adjusted for a right-sided implant.

For the femoral component, the strongest correlations between 3D translation and flexion angle occurred at the anterior flange tip (Fig. 5b, *P* < .0001), lateral posterior condyle (Fig. 5c, *P* = .0015), and medial posterior condyle (Fig. 5d, *P* = .0066). At 0°, the greatest magnitudes of displacement occurred at the medial (0.516 ± 0.392 mm) and lateral (0.550 ± 0.289 mm) fictive points. At 60°, the greatest magnitudes of displacement also occurred at the medial (0.805 ± 0.692 mm) and lateral (0.847 ± 0.632 mm) fictive points.

**Figure 6:**
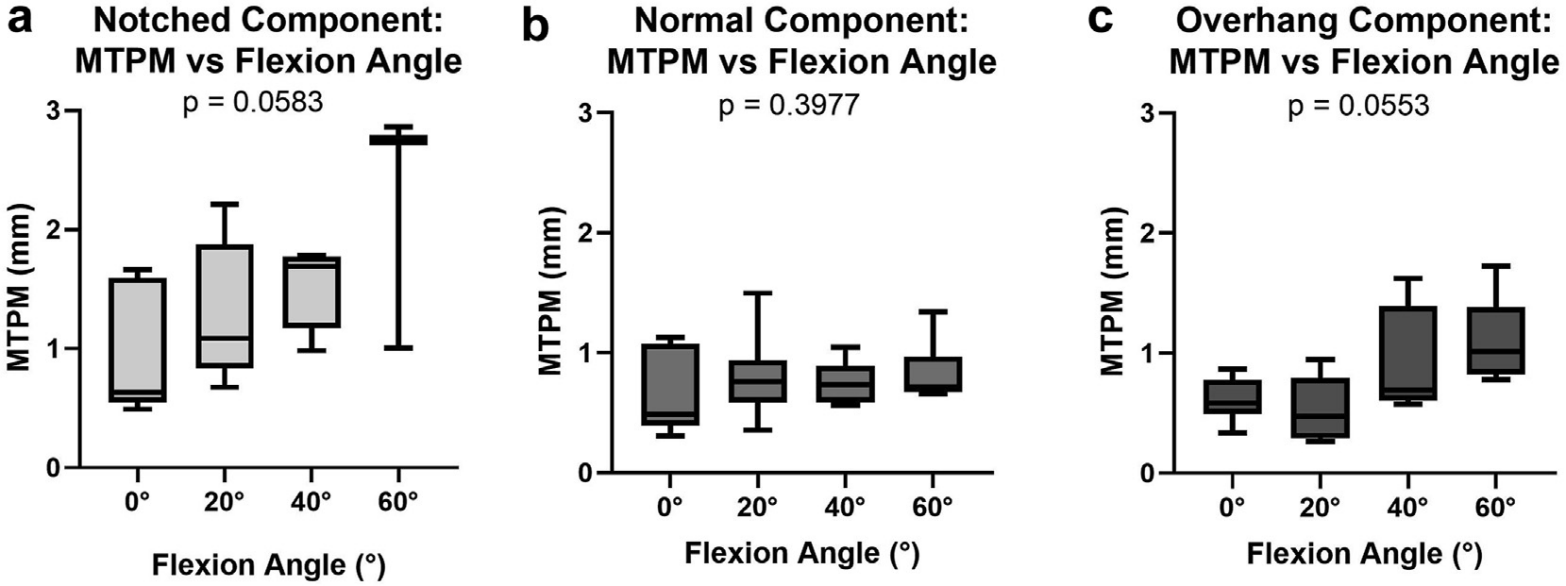
MTPM (mm) at each flexion angle for those with a notched (a), normal (b), or overhang component (c). There were no significant correlations between MTPM and flexion angle for each femoral component position.

Notched, normal, and components with overhang all had increasing MTPM with knee flexion angle. The notched components had greater MTPM at each flexion angle compared to the normal or overhang groups (Fig. 6). For the notched group, mean MTPM was 0.874 ± 0.571 mm for females and 1.148 ± 0.730 mm for males at 0°. For the overhang group, mean MTPM was 0.606 ± 0.136 mm for females and 0.608 ± 0.265 mm for males at 0°. For the normal femoral components, mean MTPM was 0.555 ± 0.314 mm for females and 0.808 ± 0.315 mm for males at 0°.

## Discussion

It has been the goal across multiple studies to understand how cementless femoral and tibial components behave under loading [19,21,[30], [31], [32]]. This study used RSA to investigate inducible displacement of cementless TKA components at multiple knee flexion angles. We found that as knee flexion angle increased, inducible displacement of both the femoral and tibial component increased. The strongest correlations were seen at the anterior flange tip for the femoral component and the stem tip for the tibial component. A subgroup analysis of the cementless femoral component found that the group with femoral notching had greater inducible displacements at deeper knee flexion angles when compared to the normal or overhang groups. Tibial components with 2 sides of underhang showed inducible displacements that were more strongly correlated to knee flexion angle than those with 1 or no sides with underhang.

To our knowledge, no clinical studies have investigated inducible displacement of cementless components at multiple knee flexion angles. There is currently no established threshold for inducible displacement to label an implant as fixed or loose; however, values as high as 1.7 mm have been reported for a well-fixed tibial component [18,33,34]. The elasticity of bone allows some displacement to occur even while components are well fixed. Laende et al. investigated the long-term migration and inducible displacements of cemented and cementless versions of a tibial monoblock design and found that the cementless version had lower displacements at 10 years postoperation, and that the displacements fell within the range expected for stable components [20]. We found our average MTPM values for the standing (0°) exams to be within this stable range for both the tibial and femoral components, with the femoral component having lower displacements than the tibial component. However, inducible displacements at 20°, 40°, and 60° of flexion were often above 1.7 mm most likely due to a greater load compared to standing at 0° knee flexion [35]. This value was established for 0° standing exams; therefore, the amount of inducible displacement at different flexion angles that would signify an unstable cementless component is unknown. However, the standing (0°) displacements are similar to those of components that have demonstrated long-term fixation, suggesting that the cementless components in this study should be predicted to have similar long-term stability.

For the tibial component, the strongest correlations between 3D translation and flexion angle occurred at the stem tip, anteromedial peg, anterolateral, and anteromedial fictive points. The maximal points of displacement occurred at the medial and lateral fictive points at 0°, while this changed to the stem tip, posteromedial, and anteromedial fictive points at 60°. All tibial components were well-fixed at 1 year postoperation; therefore, maximal displacements at the stem tip may be due to compressive forces causing motion through the tibial metaphysis, despite the components being well-fixed to the cut surface. There was a predominately posterior tilt and internal rotation of the component throughout flexion, contributing to the larger displacements of the stem tip and anteromedial fictive points at higher knee flexion angles. A study by Teeter et al assessed the same implant model with a similar cohort and found the predominant pattern of inducible displacement under loading was axial rotation with subjects having an external rotation when the knee was loaded [30]. We had similar findings at 0°; however, we noticed the components moved toward an internal rotation and posterior tilt at 60°, providing further insight into how components move as the knee starts to bend. It should be noted that the stem does not allow bone ingrowth; therefore, the maximal displacements here may be motion through the metaphysis due to compressive forces.

For the femoral component, the strongest correlations between 3D translation and flexion angle occurred at the anterior flange tip, lateral posterior condyle, and medial posterior condyle. At both 0° and 60°, the maximal displacements occurred at the medial and lateral fictive points with predominately anterior translation of the component. There was a small posterior tilt and external rotation of the femoral component at higher knee flexion angles that may explain the greater medial and lateral displacements. Han et al performed a cadaveric biomechanical study of a cementless TKA and found micromotion at posterior zones was significantly higher under stair descent load compared to walking [32]. Our results of femoral component inducible displacement found that while the strongest correlation between flexion and displacement was at the anterior flange tip, the medial and lateral fictive points, located near the most inferior part of the femoral condyles, typically had the greatest magnitudes of inducible displacement. Han et al used a cadaveric knee with standardized loads, while we had the participants in a double leg stance [32]. The participants had to hold the squat position, and this causes changes in muscle activation that may influence the results seen [36].

We observed greater femoral component inducible displacement MTPM in subjects with femoral notching at higher knee flexion angles. Several studies have investigated the impact of femoral component positioning; however, a consensus about optimal implant alignment is lacking [23]. Mahoney and Kinsey investigated the clinical consequences of femoral overhang and found overhang greater than 3 mm medially or laterally approximately doubles the odds of clinically important knee pain 2 years after TKA [37]. Marra et al found that overhang irritates the surrounding soft tissues, which could explain increased knee pain [38]. We did not measure the amount of overhang, and only assessed anterior overhang; however, we found that the overhang group had similar inducible displacements to the normal alignment group, suggesting the pain from overhang is not likely related to a lack of component fixation. Lee and Wang, and Gujarathi et al investigated the stress caused by anterior femoral notching and its possible association to periprosthetic fractures [24,26]. We found that the strongest correlation between inducible displacement and knee flexion angle was at the anterior flange tip, and the notched group had greater displacements than the normal or overhang groups. The structural integrity of the bone from notching may be compromised and could potentially cause the observed increase in inducible displacement. However, with overhang there is a possibility of increased anterior knee pain. Further investigation is needed into the impact of notching on posterior or shear forces as those were not investigated in the current analysis.

Quevedo González et al used a finite element approach and found component tilting was greater during mid-gait when shear forces would be present [12]. A combination of increased shear stresses on the tibial component with increased flexion, paradoxical anterior femoral translation in mid-flexion, and the anterior lip on the cruciate-stabilizing polyethylene liner may have resulted in more predominate tilting of the tibial component, and therefore stem tip displacement, in increased flexion, compared to relatively low amounts of stem tip displacement during standing at 0°. Quevedo González et al assessed the effect of undersizing the tibial component on the risk of bone failure [15]. They found that most load is transferred posteriorly during stair ascent and therefore suboptimal coverage of a baseplate has no apparent impact on tibial baseplate support. We found a stronger correlation between MTPM and knee flexion angle for components with both medial and lateral underhang compared to those with 1 or no sides of underhang. We also observed that components with 2 sides of underhang had lower MTPM than those with 1 or no sides of underhang. However, the differences between MTPM measurements between groups were minimal and support the results from the finite element study. Andersen et al performed a clinical study and found that undersized tibial components had high rates of migration, increased subsidence, and posterior tilt [25]. Our study only looked up to 1-year postoperation, while the study by Andersen et al assessed a different cementless implant design up to 2 years postoperation. It would be valuable to assess the longitudinal migration of this design up to 2 years postoperation; however, the current results demonstrate the stability of this cementless design and the minimal impact of an undersized component on MTPM throughout flexion.

This study has several limitations. This was an exploratory secondary analysis of a prospective cohort, and up to 15 patients were excluded for analysis due to occluded markers for RSA evaluation, leaving a small sample size for analysis. However, clinical inducible displacement data of both components throughout flexion for 24 participants is still valuable. There was a small number of participants for subgroup analyses, and further analysis will be needed to support these results. The magnitude of femoral notching or tibial underhang was not stratified for the current analysis and this may be valuable to assess in a larger study cohort. We did not measure hip–knee–ankle angles; therefore, the potential effect of coronal plane alignment was not included in the analysis. A single cementless tibial baseplate and femoral component were evaluated, and TKA devices with different features may differ in kinematics [39]. We did not standardize the joint loading during the standing position, and the load magnitude would therefore depend upon the subject’s body weight. The subjects had weight equally distributed between limbs for the standing exams, and this would create variability in how much loading went through the knee. We only performed measurements of knee flexion angles of up to 60° and therefore cannot comment on inducible displacement at deeper flexion angles. We did not investigate posterior slope in the current analysis; however, a study by Richardson et al. found that posterior slope was not associated with inducible displacement [40]. They assessed a cemented Triathlon design and future studies may be required to assess the influence of posterior slope on inducible displacement of this cementless design.

## Conclusions

We observed that both components showed greater inducible displacements at increased knee flexion angles throughout weight-bearing flexion. The strongest correlation was observed at the anterior flange tip of the femoral component and the stem tip of the tibial component, indicating tilting was occurring. These findings suggest that inducible displacements throughout weight-bearing flexion are due to mechanical loading and demonstrates that this cementless design remains well-fixed throughout weight-bearing flexion.

## CRediT authorship contribution statement

**Rebecca A. Hext:** Writing – review & editing, Writing – original draft, Visualization, Investigation, Formal analysis, Data curation. **Jordan S. Broberg:** Writing – review & editing, Methodology, Formal analysis, Data curation, Conceptualization. **James L. Howard:** Writing – review & editing. **Brent A. Lanting:** Writing – review & editing. **Matthew G. Teeter:** Writing – review & editing, Conceptualization.

## Appendix A Supplementary data

Conflict of Interest Statement for Broberg

Conflict of Interest Statement for Hext

Conflict of Interest Statement for Lanting

Conflict of Interest Statement for Howard

Conflict of Interest Statement for Teeter
