# Supplementary material for: Inducible Displacement of Cementless Femoral and Tibial Components Throughout Weight-bearing Flexion
Source: Arthroplast Today. 2025 Jun 4;33:101723. doi: 10.1016/j.artd.2025.101723 (PMC12172991; doi:10.1016/j.artd.2025.101723)
Supplement: Conflict of Interest Statement for Howard [file mmc4.pdf]

CONFLICT OF INTEREST STATEMENT

American Association of Hip and Knee Surgeons

(Adopted from the American Academy of Orthopaedic Surgeons disclosure statement)

The following form **must be filled out completely and submitted by each author (example, 6 authors, 6 forms). All items require a response. If there is no relevant disclosure for a given item, enter "*None*."**

Manuscript Title: Inducible Displacement of Cementless Femoral and Tibial Components Throughout Weightbearing Flexion

1. Royalties from a company or supplier (The following conflicts were disclosed)

   None

2. Speakers bureau/paid presentations for a company or supplier (The following conflicts were disclosed)

   Paid presenter or speaker for DePuy, A Johnson & Johnson Company, Intellijoint, Sanofi-Aventis, Smith & Nephew, Stryker, and Zimmer. All are unrelated to the present study.

3A. Paid employee for a company or supplier (The following conflicts were disclosed)

   None

3B. Paid consultant for a company or supplier (The following conflicts were disclosed)

   Paid consultant for Zimmer, Stryker, Smith & Nephew, Intellijoint, and DePuy, A Johnson & Johnson Company. Fees are unrelated to the present study.

3C. Unpaid consultants for a company or supplier (The following conflicts were disclosed)

   None

4. Stock or stock options in a company or supplier (The following conflicts were disclosed)

   None

5. Research support from a company or supplier as a Principal Investigator (The following conflicts were disclosed)

   Research support from DePuy, A Johnson & Johnson Company, and Stryker. The only applicable one for the current study is Stryker, however the fees are unrelated to the present study.

6. Other financial or material support from a company or supplier (The following conflicts were disclosed)

   Other support from DePuy, A Johnson & Johnson Company, Microport, Smith & Nephew, Stryker, and Zimmer. Fees are unrelated to the present study.

7. Royalties, financial or material support from publishers (The following conflicts were disclosed)

   None

8. Medical/Orthopaedic publications editorial/governing board (The following conflicts were disclosed)

   None

9. Board member/committee appointments for a society (The following conflicts were disclosed)

   None

Each author must sign AND print or type his/her name, date and submit a separate form
In addition, one BLINDED Conflict of Interest form (no author names used) should be submitted per manuscript with all author disclosures.

Howard | [signature] | Oct 7/24

Author Name (Print or Type) | Author Signature | Date
